# A Novel Point-of-Care Rapid Diagnostic Test for Screening Individuals for Antibody Deficiencies

**DOI:** 10.1007/s10875-021-01179-0

**Published:** 2021-11-27

**Authors:** Shirli Israeli, Allison Golden, Melissa Atalig, Najla Mekki, Afef Rais, Helen Storey, Mohamed-Ridha Barbouche, Roger Peck

**Affiliations:** 1 Diagnostics, PATH, Seattle, WA USA; 2 Department of Immunology, Pasteur Institute of Tunis, Tunis, Tunisia; 3 University of Tunis El-Manar, Tunis, Tunisia

**Keywords:** Point of care, Primary antibody deficiency, Primary immunodeficiency disease, Rapid diagnostic test, Screening, Vaccine-derived poliovirus

## Abstract

**Purpose:**

No rapid diagnostic test exists to screen individuals for primary antibody deficiencies (PAD) at or near the point of care. In settings at risk for polio where live oral polio vaccine is utilized, undiagnosed PAD patients and cases with delayed diagnosis constitute a potential reservoir for neurovirulent polioviruses, undermining polio eradication.

This research aimed to develop a rapid screening test suited for use in resource-limited settings to identify individuals with low immunoglobulin G (IgG) levels, enabling early diagnosis and appropriate treatment.

**Methods:**

Three prototype tests distinguishing low and normal IgG levels were evaluated with a blinded panel of serum/plasma specimens from 32 healthy controls and 86 primary immunodeficiency-confirmed patients with agammaglobulinemia, common variable immunodeficiency, and hyper-IgM syndrome, including 57 not receiving IgG therapy. Prototype tests were compared to laboratory reference and clinical case definition.

**Results:**

The leading prototype correctly identified 32 of 32 healthy controls. Among primary antibody deficiency patients not receiving IgG treatment, 17 of 19 agammaglobulinemia, 7 of 24 common variable immunodeficiency, and 5 of 14 hyper-IgM were correctly identified by the prototype, with 67% agreement with the reference assay.

**Conclusion:**

The Rapid IgG Screen (RIgGS) test can differentiate between low IgG levels associated with agammaglobulinemia and normal IgG antibody levels. Differentiating CVID and hyper IgM was challenging due to the wide range in IgG levels and influence of high IgM. This test can facilitate the identification of patients with primary antibody deficiencies and support polio surveillance initiatives.

**Supplementary Information:**

The online version contains supplementary material available at 10.1007/s10875-021-01179-0.

## Introduction

Inborn errors of immunity also known as primary immunodeficiency diseases (PIDs) are a heterogeneous group of more than 400 genetic disorders caused by mutations in genes involved in the development and/or the function of the immune system. PID prevalence around the world ranges from 0.81 to 30.5 per 100,000 population [1]. The higher prevalence is observed in populations characterized by a high rate of consanguinity [1, 2]. Primary antibody deficiencies (PADs) [3] are among the most common PIDs. They are characterized by an inability to generate sufficient antibody levels needed to induce a protective immune response. Patients with PAD are prone to recurrent bacterial infections particularly in the respiratory and gastrointestinal tracts. They are also at risk of contracting viral infections including with live viral vaccines.

Approximately 150 countries still use the oral poliovirus vaccine (OPV) as part of their polio eradication effort [4].

The WHO has initiated a guideline to detect excretors of poliovirus among PID patients upon receiving OPV or in close contact with someone recently vaccinated, referred to as immunodeficiency-related vaccine-derived polioviruses (iVDPVs). In addition to the risk of developing paralytic poliomyelitis, individuals infected with iVDPV present the potential risk of initiating vaccine-derived poliovirus (VDPV) outbreaks.

Children with low immunoglobulin G (IgG) levels who are given OPV are at an approximately 3,000-fold increased risk for shedding the virus [5]. Prolonged viral replication and shedding can result in mutations of the attenuated virus used in OPV into a VDPV capable of circulating in underimmunized populations. The capacity for neurovirulence of these vaccine-derived strains may be similar to that of the wild virus it is intended to protect against. Addressing PADs in polio surveillance is essential for the eradication effort [4, 6].

No rapid diagnostic test (RDT) is currently available to screen individuals for PADs at the point of care in low- and middle-income countries. Initial suspicion of PID is based on warning signs [7] and may result in recommendation for a patient referral to immunology specialists for follow-up testing, which commonly includes laboratory testing for antibody levels [8]. Such follow-up investigations require infrastructure and may not reach remote, at-risk, and undiagnosed populations. In response, PATH has developed a simple, easy-to-use, prototype to screen for PADs, Rapid IgG Screen (RIgGS). The test is designed as a quick screening tool for primary healthcare workers to detect low antibody levels in patients with observed clinical symptoms. The test is intended to meet the needs of limited-resource settings, particularly for point-of-care screening and to benefit polio surveillance programs.

Research and development was guided by literature reviews and stakeholder interviews that informed product specifications. A key design target was to determine an IgG threshold level indicative of PAD to serve as a visible threshold point for the assay. Other key specifications included test result in less than 20 min; ability to use fingerstick blood, plasma, or sera; and a simple workflow that does not require power or instrumentation.

Three versions of RIgGS prototypes were developed at PATH and then evaluated at Pasteur Institute of Tunis, using a panel of stored specimens from a plasma/sera bank. This collection included healthy and PID-confirmed individuals prior to or currently undergoing intravenous immunoglobulin treatment. The major causes of PAD were targeted—i.e., agammaglobulinemia (AG), common variable immunodeficiency (CVID), and hyper-IgM syndrome (HIGM) [3]. The samples were blinded to the laboratory technician evaluating the test and to the team that analyzed the results. Only when all the tests were run, and the results were interpreted as at risk for PAD or normal levels of IgG were the true status of the samples revealed.

## Methods

### Components in the of RIgGS Prototypes

**Fig. 1:**
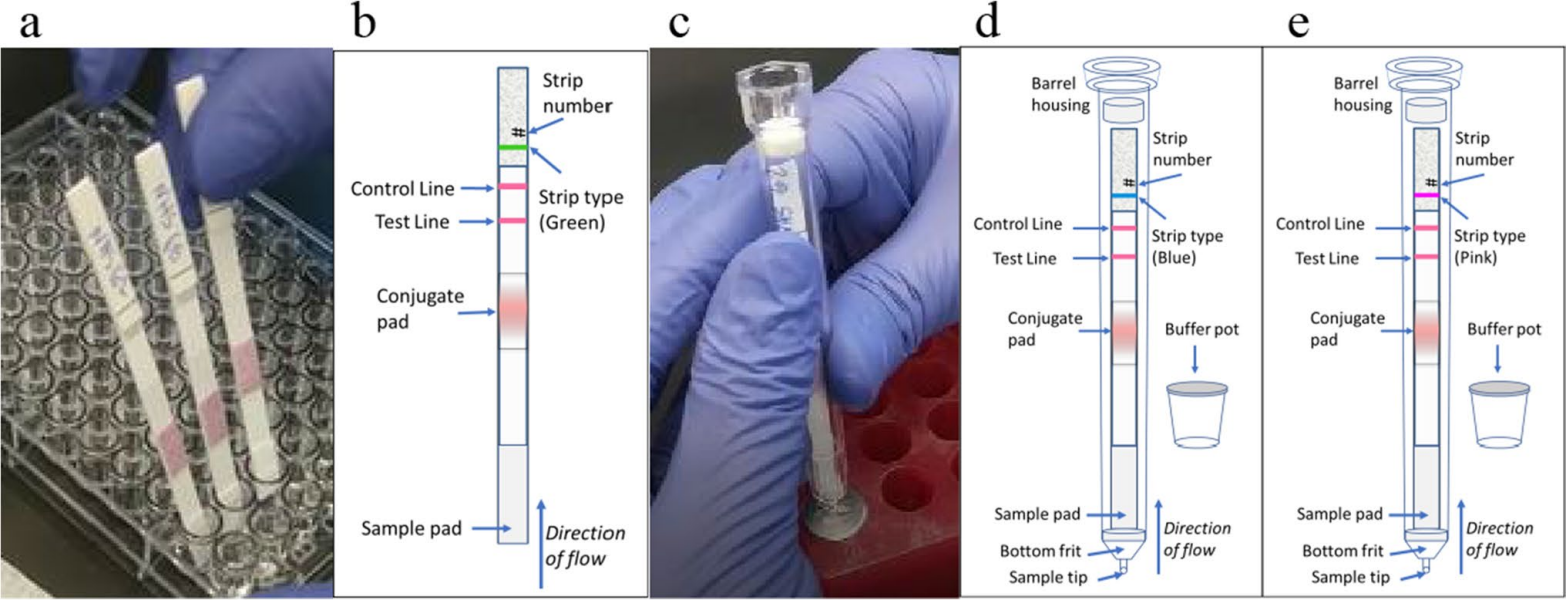
Depiction of prototype tests under evaluation. **a** Basic strip in a well; **b** basic strip diagram, green prototype; **c** barrel-integrated housing; **d** barrel-integrated housing diagram for the blue and 1e barrel-integrated housing diagram for the pink prototypes. The green, blue and pink prototypes differ in the amount of protein stripped on the membrane

PATH constructed the prototype RIgGS as lateral flow test strips. Combinations of Protein L (PRO-1790, ProSpec, Rehovot, Israel), Protein A (IA000019-10–2001-0 M rSPA native recombinant *S. aureus* Protein A [animal free], Syd labs, Hopkinton, Massachusetts, USA), and human immunoglobulin G (16–16-090,707, Athens Research & Technology, Inc, Athens, Georgia, USA) were applied onto nitrocellulose membranes (90CNPH-N-SS40 and CNPC-SS12, MDI, Ambala Cantt, India) using a contact tip dispenser (Model XYZ3060, BioDot, Irvine, CA, USA). The lower membrane is 90CNPH-N-SS40 and is striped with one line of 2 mg/ml Protein A (blue prototype at 0.65 ul/cm, pink prototype at 0.55 ul/cm, and green prototype at 0.9 ul/cm) and 5 lines of 0.5 mg/ml Protein L (blue prototype at 0.65 ul/cm, pink prototype at 0.55 ul/cm, and green prototype at 0.8 ul/cm). The upper membrane is CNPC-SS12 and is striped with a test line of Protein L (0.25 mg/ml, 0.8 ul/cm) and procedural control line of human IgG (1.5 mg/ml, 0.8 ul/cm). A detection conjugate was prepared from 40 nm colloidal gold particles with adsorbed Protein A and applied to non-woven conjugate pad materials (PTR7, MDI). Test strips were prepared using the nitrocellulose and conjugate pad materials combined with GE Healthcare LF1 sample separation pad (LF1, 8121–6621, GE Healthcare, Marlborough, MA, USA), absorbent pads (R025 and 243, Ahlstrom, Helsinki, Finland), cover tape (7759, Adhesives Research, Glen Rock, PA, USA), and adhesive-laminated polystyrene backing cards (GL-57623, Lohmann, Orange, VA, USA). Test strips were cut into 5 mm strips using a Kinematic Matrix 2360 cutter. The test running buffer was prepared using phosphate-buffered saline with Tween-20 (Sigma-Aldrich P3563, St. Louis, MO, USA). Barrel-shaped housings with an integrated sample collection tip and prefilled foil-sealed running buffer pot were sourced from BioSure (Nazeing, Essex, England). One prototype was a basic strip test without a cassette housing (Figs. 1a, b), while two prototypes used barrel-integrated housings, assembled by placing test strips into them (Figs. 1c, d, e). The two barrel-integrated housing tests differed by slight variations in the test formulation to achieve the targeted threshold. To run the basic strip prototype, the strips were placed into flat-bottom multiwell plates (29,442–070 Corning 9017, Tewksbury, MA, USA) to draw up sample and buffer in the well by capillary action.

### Design of the RIgGS Prototypes

The sample mixed with running buffer enters the sample pad and then traverses the lower membrane by capillary action where it interacts with Protein A and Protein L. This first interaction is intended to reduce by sequestration the concentration of IgG in the sample based on the chosen threshold. If the concentration of IgG in the sample is below the chosen threshold, no IgG will progress to the rest of the test. If the concentration of IgG in the sample is higher than the chosen threshold, free IgG will continue up the strip to the conjugate pad. Free IgG binds the Protein A gold conjugate and travels up the strip to the upper membrane. The upper membrane contains the test line and control line. Only conjugate that has formed a complex with IgG will bind with the Protein L test line; free Protein A gold conjugate will bind the control line. The three prototypes differ by the quantity of striped Protein A and Protein L on the lower membrane. This allowed us to evaluate RIgGS prototypes with slightly different thresholds.

### Developing Test Threshold

Three candidate prototypes were developed to detect approximately 3 g/L IgG, which was targeted as the initial threshold for identifying children at risk for PAD based upon literature review [9–11]. Research and development were conducted using commercially available serum specimens with varying concentrations of IgG but not necessarily from PID patients. These thirty-three specimens were acquired from Discovery Life Sciences (Los Osos, CA, USA) with IgG concentration characterized by Siemens BN II System nephelometer (Siemens Healthcare GmbH, Erlangen, Germany). IgG levels in the chosen samples ranged from 0.12 to 5.76 g/L. No data were available for patient diagnosis, preexisting conditions, patient treatment, and IgA or IgM concentration.

### Clinical Samples

Collection, characterization, and storage of the specimens used in this study were conducted within an ethically approved clinical research study reviewed and approved by Comité d’Ethique Médicale, Institut Pasteur de Tunis (IRB protocol number IPT/11/LR11IPT02, IPT/PCI/LR11IPT02, and IPT/23/I/LR11IPT02).

PATH’s Research Determination Committee reviewed this study and determined that it did not involve human subjects as defined in 45 CFR 46.102(e). The use of the stored specimens in this study was further confirmed to be acceptable as secondary research and within consent given by the patients and families.

One hundred and eighteen stored samples were blinded for the PAD diagnosis and prior immunoglobulin testing for the prototype evaluation and parallel reference assay testing (turbidimetry, SPAPLUS®, Binding Site, Birmingham, UK). The samples included 32 healthy donors and 86 PAD patients for whom a diagnosis of AG, CVID, and HIGM had been assigned according to the criteria of the Expert Committee of International Union of Immunological Societies [12]. These PAD included 57 patients prior to receiving treatment (*n* = 19, 24, and 14, respectively) and 29 undergoing intravenous immunoglobulin treatment (*n* = 8, 16, 5, and respectively) (Table 1).

**Table 1:** Samples included in study, from individuals confirmed to have PAD or healthy controls

**Diagnosis**	*Not receiving treatment for PID*		*Receiving treatment for PID*	
	*Number of individuals*	*Age range*	*Number of individuals*	*Age range*
**AG**	19	20 days–11.5 years	8	1 month–13 years
**CVID**	24	9 months*–50 years	16	15 months*–54 years
**HIGM**	14	11 months–38 years	5	5 months–3 years
**Healthy control**	32	3 months–14 years	n/a	n/a

*AG* agammaglobulinemia; *CVID* common variable immunodeficiency; *HIGM* hyper-IgM syndrome; *n/a* not applicable

^*^For CVID patients under 4 years, diagnosis was first suspected and then confirmed through follow-up

### Evaluation of Prototypes at Institut Pasteur de Tunis

Laboratory technicians were trained to correctly use the RIgGS prototypes, including reading and interpreting negative and positive results and observing test run characteristics. Each specimen was run on all three prototypes, with the basic strip prototype coded as green and the two barrel-integrated housing prototypes coded as blue and pink (Fig. 1). The prototype testing was conducted on the same day, by the same laboratory technician. Reference testing for quantitative IgG, IgA, and IgM concentrations was conducted in parallel to prototype testing. The reference method (turbidimetry, SPAPLUS, Binding Site) used was the standard of care for patients for determining immunoglobulin levels. Test users remained blinded to reference results until completion of all sample testing.

### Procedure to Run Specimens with the Basic Strip Prototype

Green prototype tests were run by first vortexing to mix the sample (serum/plasma) for approximately 5 s. Then 1.5 µl of sample and 10 µL test running buffer were combined in a microplate well and mixed by pipetting at least 10 times. The strip was placed into the well with the diluted sample for 90 s. Then, the strips were moved into a new well with 90 µL of test running buffer. Results were read at 15 min.

### Procedure to Run Specimens with the Barrel-Integrated Housing Prototypes

Blue and pink prototype tests were run by first vortexing to mix the sample (serum/plasma) for approximately 5 s. Then 1.5 µl of sample was added into the capillary sample tip at the bottom of the barrel, making sure that the full sample volume was dispensed into the tip. The barrel tip was then slowly pushed into the buffer pot with a smooth, steady motion, piercing the buffer pot foil seal and sealing the barrel into the pot in an upright position. The test remained standing vertical in the buffer pot for the duration of the test. Results were read at 15 min.

### Interpretation of Results and Quality Control

Figure 1 shows the two methods. At the designated time, the laboratory technician visually read the prototypes. The presence of a control line was required for the test to be considered valid and interpreted. The presence of any line of any intensity at the test line location was interpreted as reactive, indicating an IgG level above the test visible threshold. Test line intensity was scored using a color intensity guide. The absence of any line at the test line location was interpreted as nonreactive, indicating an IgG level below the test threshold (i.e., at risk for PAD). Quality of the test runs was assessed through observations by test operators and compared to predetermined criteria established prior to the study. The primary criterion for run quality was a valid result indicated by the presence of a control line.

Secondary criteria for repeating tests included monitoring for improper sample or buffer flow in the test. These criteria included observation of excessive time for capillary flow (as measured by time for visible fluid to reach specific sections of the test) and observation of fluid movement within the barrel that did not transfer to the test strip. Tests were repeated if the results were not valid (no control line), and a portion of tests were repeated for secondary quality criteria. The first valid result for a sample was used for data analysis, unless repeated. Repeated test results were used so long as they were valid and secondary test run criteria were met better than the first run.

### Statistical Analysis of Analytical Performance

To compare the target threshold to the analytical performance with clinical specimens, the data for the blue prototype was modelled as a continuous function of probability of detection versus IgG concentration for the study specimens. Due to interference of excess IgM, results obtained from HIGM specimens were not included in the model. The logistic regression model was fitted using a Hamiltonian Monte Carlo procedure implemented in Stan via the brms package in software R [13, 14]. The test binary result (0, 1) was the dependent variable, and the untransformed IgG concentration was the independent variable. Informative Gaussian priors with a mean of 0 and standard deviation of 3 were used for the model parameters. Two chains were each run for 10,000 iterations after a burn-in of 5,000 iterations. Convergence was visually assessed from the plots of both parameters. The fitted line and shaded area were plotted to identify median and the 95% credible interval from the logit transformed posterior distributions from the linear predictor.

## Results

Evaluation of RIgGS performance using characterized clinical specimens: healthy controls, agammaglobulinemia, common variable immunodeficiency, and hyper-IgM:

### Healthy Controls

**Fig. 2:**
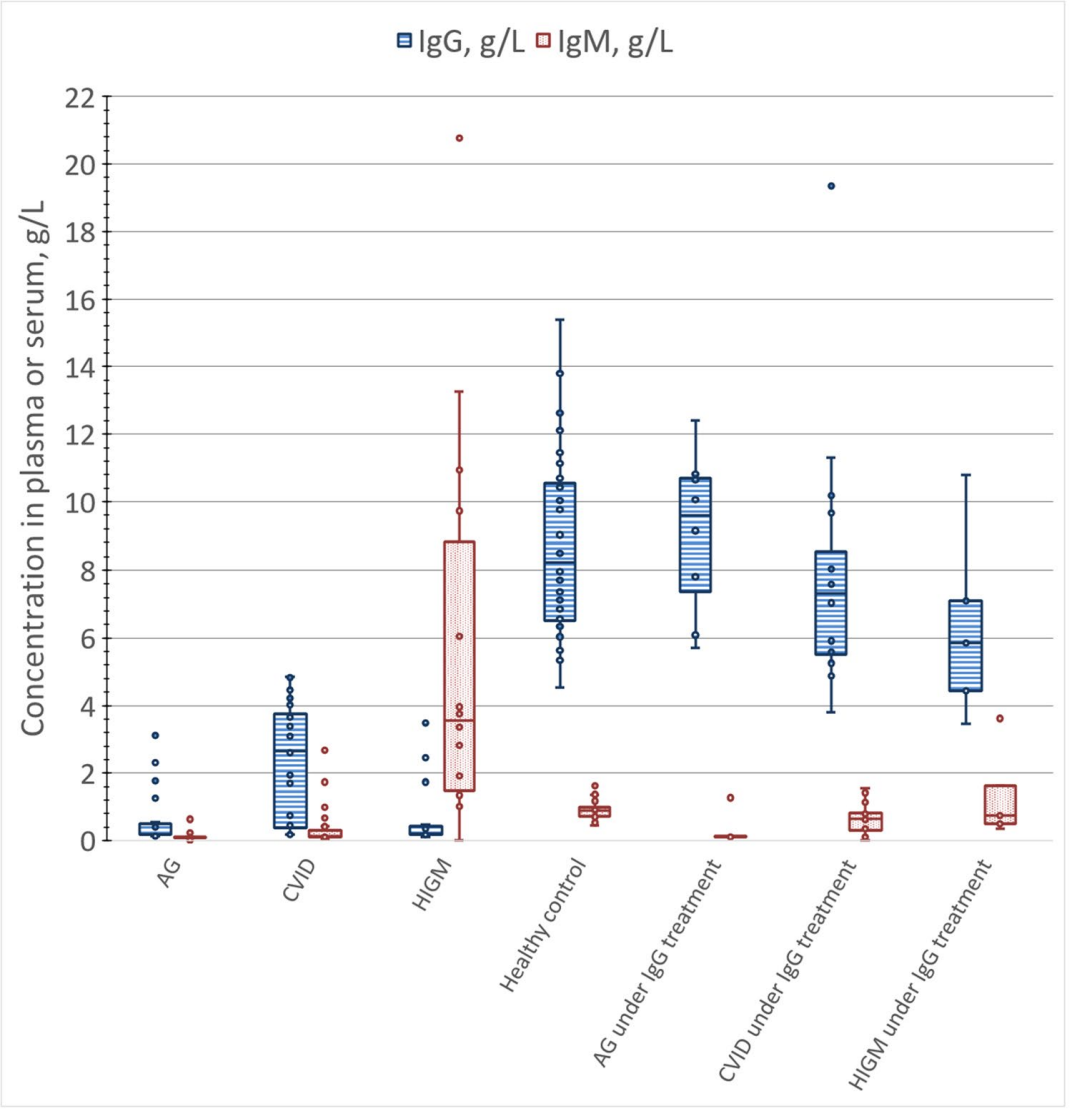
Distribution of IgG and IgM in study samples, as measured by reference assay. Box plot of first- to third-quartile IgG and IgM concentrations are shown, with inclusive median indicated within box by horizontal line

**Fig. 3:**
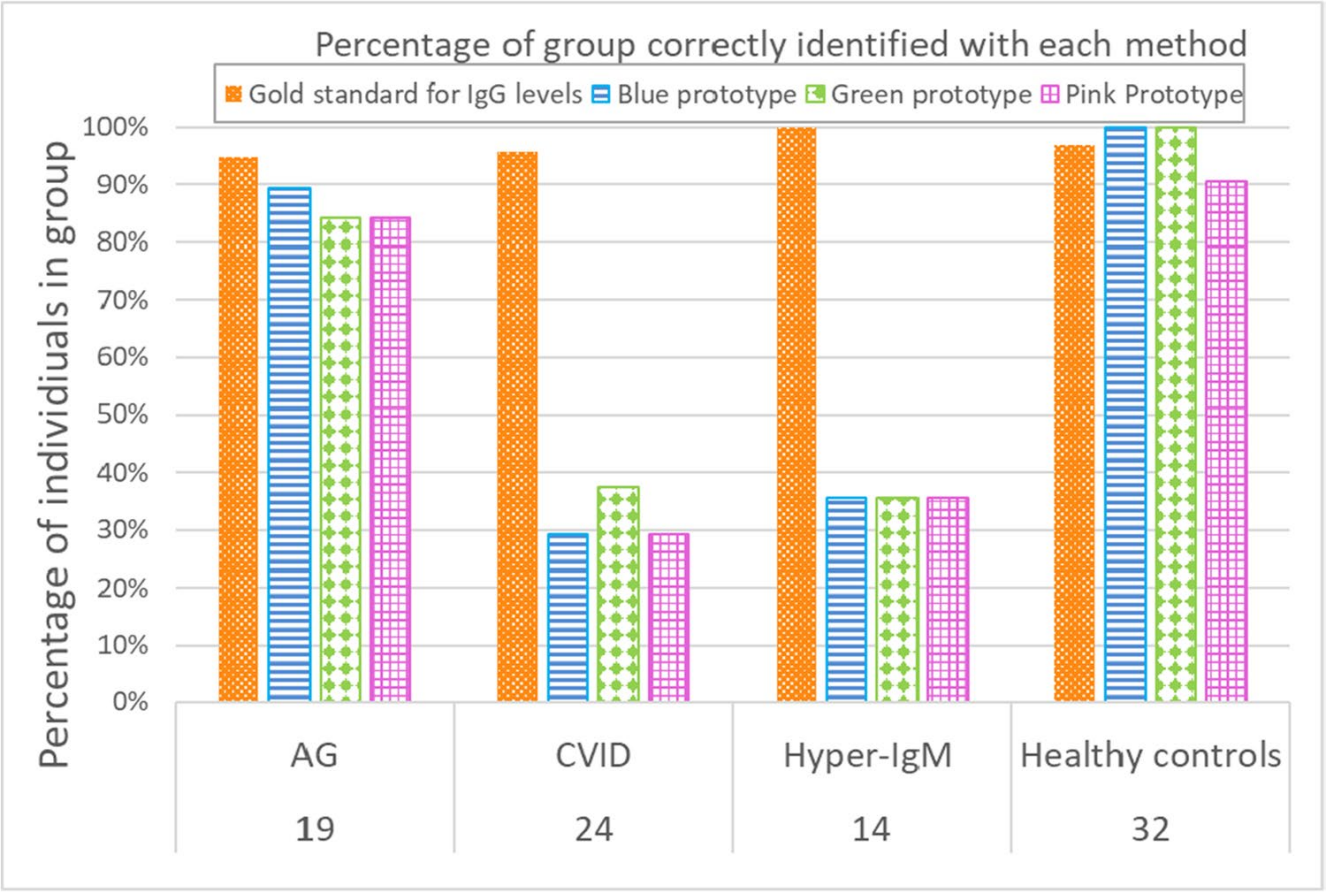
Percentage of samples identified correctly by the reference assay and the blue, green, and pink prototypes as either at risk with low IgG levels or not at risk with normal IgG levels from individuals not receiving treatment in the subgroups AG, CVID, HIGM, and healthy controls. Correct identification was defined by comparison to the diagnostic confirmatory test for the PAD subtype as at risk for low IgG levels and healthy controls expected to have normal IgG levels

The study included 32 individuals identified as healthy controls. The IgG reference test results ranged from 4.5 to 15.4 g/L (Fig. 2). Blue, green, and pink prototypes were reactive 100% (32 of 32), 100% (32 of 32), and 90.6% (29 of 32), respectively, in this category (Fig. 3).

### Agammaglobulinemia

The study included 19 individuals identified as AG and not yet receiving intravenous IgG substitutive treatment. The IgG reference test results ranged from 0.1 to 3.1 g/L (Fig. 2). Blue, green, and pink prototypes were nonreactive 89.5% (17 of 19), 84.2% (16 of 19), and 84.2% (16 of 19), respectively, in this category (Fig. 3).

The study included 8 individuals identified as AG receiving intravenous IgG treatment. The IgG reference test results ranged from 5.7 to 12.4 g/L (Fig. 2). Blue, green, and pink prototypes were reactive 100% (8 of 8), 100% (8 of 8), and 100% (8 of 8), respectively, in this category (data not shown).

### Common Variable Immunodeficiency

The study included 24 individuals identified as CVID and not yet receiving intravenous IgG substitutive treatment. The IgG reference test results ranged from 0.2 to 4.8 g/L (Fig. 2). Blue, green, and pink prototypes were nonreactive 29.2% (7 of 24), 37.5% (9 of 24), and 29.2% (7 of 24), respectively, in this category (Fig. 3).

The study included 16 individuals identified as CVID receiving intravenous IgG substitutive treatment. The IgG reference test results ranged from 3.8 to 19.4 g/L (Fig. 2). Blue, green, and pink prototypes were reactive 87.5% (14 of 16), 100% (16 of 16), and 93.8% (15 of 16), respectively, in this category (data not shown).

### Hyper-IgM

The study included 14 individuals identified as HIGM and not yet receiving intravenous IgG substitutive treatment. The IgG reference test results ranged from 0.1 to 3.5 g/L (Fig. 2) and IgM ranged from 1.0 to 20.8 g/L, whereas the upper end of the normal IgM reference interval in the literature is 2.9 g/L [15]. Blue, green, and pink prototypes were nonreactive 35.7% (5 of 14), 35.7% (5 of 14), and 35.7% (5 of 14), respectively, in this category (Fig. 3).

The study included 5 individuals identified as HIGM and receiving intravenous immunoglobulin treatment. The IgG reference test results ranged from 3.5 to 10.8 g/L (Fig. 2). Blue, green, and pink prototypes were reactive 100% (5 of 5), 100% (5 of 5), and 100% (5 of 5), respectively, in this category (Fig. 2).

### Reference and Prototype Agreement

A single prototype was chosen for data analysis, based on both highest agreement with reference and preferred form-factor which was the blue prototype. Analysis focused on healthy controls and PAD cases not receiving immunoglobulin treatment, as would be encountered in screening. Agreement between the reference assay result and the blue prototype for clinical case definitions of healthy control, and AG, CVID, and HIGM not receiving treatment was 96.9% (31 of 32), 84.2% (16 of 19), 33.3% (8 of 24), and 35.7% (5 of 14), respectively, while total agreement across all case definitions was 67.4% (60 of 89) (Table 2).

**Table 2:** Percentage of agreement between the reference assay result and blue prototype

	Agreement Low Ref Low test^1^	Agreement Normal Ref Normal test^2^	Disagreement Low Ref Normal test^3^	Disagreement Normal Ref Low test^4^	Percent agreement
Healthy control	0	31	1	0	96.9
Agammaglobulinemia before treatment	16	0	2	1	84.2
CVID before treatment	7	1	16	0	33.3
Hyper-IgM before treatment	5	0	9	0	35.7
Total	28	32	28	1	67.4

^1^Reference assay sample result below minimum normal IgG value, prototype nonreactive (IgG not detected). ^2^Reference assay sample result above minimum normal IgG value, prototype test line reactive (IgG detected). ^3^Reference assay sample result below minimum normal IgG value, prototype test line reactive (IgG detected). ^4^Reference assay sample result above minimum IgG value, prototype nonreactive (IgG not detected)

**Fig. 4:**
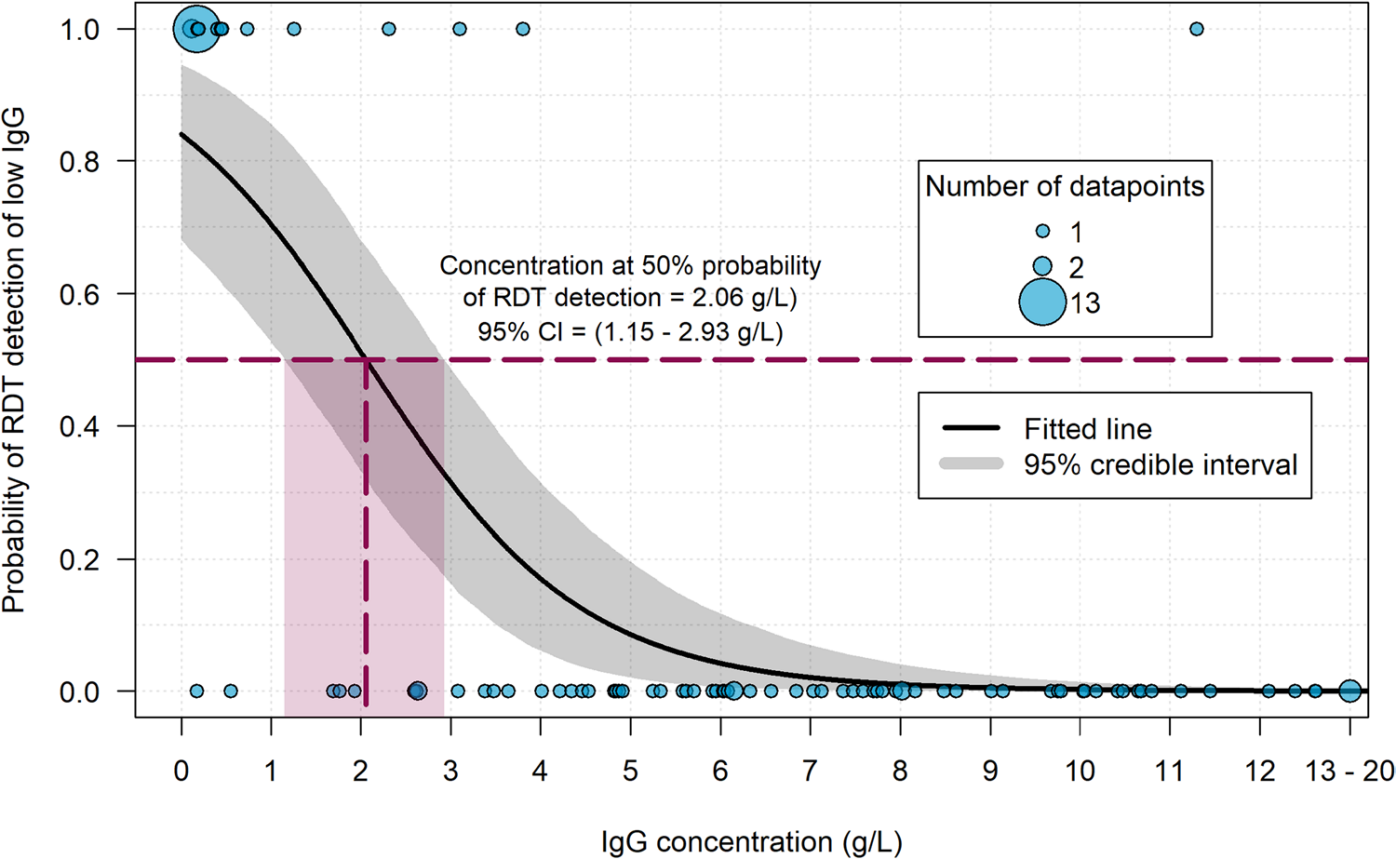
Common variable immunodeficiency IgG levels (before treatment) versus age of patient when sample collected (blue dots). Lower bound of normal IgG levels by age is indicated by the red line using the reference test for IgG levels. The bold black line is targeted 3 g/L threshold

The IgG reference test value was also compared to the age-related reference interval of normal IgG (supplemental file). Samples with reference test values below the normal range were identified as having low IgG. Using these criteria, the IgG reference test agreement with the clinical case definitions of healthy controls and PID subtypes not under treatment of AG, CVID, and HIGM was 96.9% (31 of 32), 94.7% (18 of 19), 95.8% (23 of 24), and 100% (14 of 14), respectively. Ten of the 24 specimens with CVID had a reference IgG value above the RDT prototype targeted threshold of 3 g/L. There was no correlation between the patient age and their IgG reference value (Fig. 4).

### Logistic Regression Model: Threshold Analysis

With a large range of individual concentrations tested and because rapid tests may have equivocal results near the detection threshold, one way of modeling detection thresholds is to fit a continuous curve to the positivity data to identify at what concentration the test is expected to be positive. The transition point on this curve where ~ 90% of tests are expected to show signal then can be identified as the resulting detection threshold. This fitting and model of the IgG detection threshold was conducted using all samples except those representing HIGM, given the likelihood of interference by the IgM (*n* = 99). The test was designed with a target threshold of 3 g/L IgG using a panel of specimens with known IgG values. However, logistic regression model using the study data from the blue prototype as compared to the reference assay IgG results for all sample types showed that the threshold achieved with the test was approximately 2 g/L with the study samples.

## Conclusion

Out of the three prototypes evaluated, the green and blue prototypes performed well with specimens from healthy control individuals and from patients with cases of AG where the IgG concentration was low. The blue prototype had preferred usability characteristics for a point-of-care test, such as not requiring sample dilution, pre-measured buffer, and an integrated sample collection and test housing, as compared to the bare test strip form of the green prototype. Because of this preference, the blue prototype was used in full analysis.

With overall 67% agreement with the reference assay, the blue prototype could differentiate between low IgG levels associated with agammaglobulinemia and normal IgG antibody levels. Disagreement was highest with elevated IgM levels and higher IgG levels in CVID, indicating that agreement was immunoglobulin concentration-dependent throughout all specimen types.

The test was designed with a target threshold of 3 g/L IgG using commercially available serum specimens with known IgG values. However, logistic regression model showed that the threshold achieved with the test was approximately 2 g/L with the study samples (Fig. 5). This points to a limitation in use of such commercial samples for development given that IgA and IgM values were not provided with the samples, which also were not necessarily from PID patients. Future iterations of the prototypes can be adjusted in formulation in order to reach the initial target threshold of 3 g/L IgG with true PID samples or to target another threshold based on ongoing interviews with immunology experts, and such further development will more precisely characterized samples and relevant specimens for further development.

The study included 24 patients with CVID (not yet receiving intravenous IgG substitutive treatment) that had IgG levels ranging from 0.2 to 4.8 g/L. The blue prototype identified 7 out of the 24 patients. By increasing the threshold to 3 g/L IgG, the test would still miss 10 out of the 24 patients (Fig. 4). While further increasing the threshold may increase the risk of a false positive of identifying the risk of PAD in individuals with near-normal antibody levels or without any underlying antibody deficiency, a positive test screening will trigger additional immunological investigations which may resolve any false positives that occur. Higher sensitivity with a potential tradeoff in specificity may be preferred for a screening test in which identifying individuals in need of follow-up investigation is necessary for the health of the patient and community.

The prototypes performed poorly with HIGM patient samples. The results indicated that the excess IgM was likely interacting with the test similarly to IgG. Patients with HIGM have reported values up to 34.84 g/L, more than ten times the concentration in healthy patients [16]. The prototypes were not designed with specifications for high IgM tolerance in mind, and this evaluation has highlighted the importance of this criterion. Future iterations of the prototypes will be redesigned to mitigate for elevated IgM levels to be able to detect low IgG levels in HIGM specimens.

Future development of the RIgGS blue prototype should focus on honing the assay threshold and mitigating effect of HIGM specimens. The assay threshold should be adjusted to the intended target of 3 g/L by changing the Protein A and Protein L concentrations on the lower membrane, increasing the binding of IgG in the sample. The test was design in a way that the threshold can be modified in response to the prototype performance and the intended use of the test. Mitigating the high levels of IgM found in HIGM specimens could be achieved through several mechanisms. Possible approaches include utilizing anti-IgM in the lower membrane, changing the test line to a more specifically bind anti-IgG, or changing the ratios of the Protein A and Protein L in the lower membrane.

Some limitations of this study prevent a robust performance verification for screening of PAD. The study was conducted with frozen plasma and serum samples, not whole blood. While the test has been designed for use with whole blood from fingerstick, a number of other interfering substances could be present in individual blood samples which could impact both the readability of the test and the result itself. Effect of hematocrit, and endogenous and exogenous interferences such as hemolysis or lipemia need to be tested prior to RIgGS becoming a commercially available test. Antibody levels can vary widely between PID subtypes, and while all types included were expected to have subnormal levels of IgG by age, agreement of the prototype tests to reference assay varied widely with respect to type of PID, and stratification for the purpose of performance would be limited by numbers represented in each subtype category. Additionally, this study did not analyze the levels of immunoglobulin A with respect to the test agreement, given that it was typically much lower in concentration relative to IgG levels and to the IgM levels in the HIGM group. IgA is important to mucosal immunity, and average values were lower in PID subtypes as compared to healthy controls, which could have some additional influence on the identification of low IgG levels. One limitation in the interpretation of the threshold analysis as compared to the results in development based on design targets was that the panel of samples used for development was not subjected to the same analysis as the clinical samples.

Currently, antibody testing as a preliminary step is conducted in laboratory settings that require infrastructure. As a result, the testing does not often reach remote, underserved populations. PATH’s RIgGS prototype is intended to augment the standard of care by introducing a rapid test to detect low IgG levels that can be conducted in primary health care settings, earlier in the patient journey. Similar to rapid tests for other diseases, it was designed to meet the needs of limited-resource settings, particularly at the point of care, where easy-to-use, affordable tests are most needed.

Our RIgGS has the ability to inform healthcare providers who can then refer patients with a positive result to more advanced and specialized facilities. Rapid screening to identify individuals at risk for PAD should prevent or stop OPV being given to those with suspected PAD, inform surveillance of vaccine-derived poliovirus, and facilitate efforts to control ongoing shedding. With increased precision, RIgGS for PAD have the potential not only as a useful tool toward solving the polio eradication equation but to help mitigate suffering from an inherited condition.

## Supplementary Information

Below is the link to the electronic supplementary material.

Supplementary file1 (XLSX 19 KB)

## Data Availability

All data generated or analyzed during this study are included in this published article and its supplementary information files.
